# Penetrance estimates for *BRCA1 *and *BRCA2 *based on genetic testing in a Clinical Cancer Genetics service setting: Risks of breast/ovarian cancer quoted should reflect the cancer burden in the family

**DOI:** 10.1186/1471-2407-8-155

**Published:** 2008-05-30

**Authors:** D Gareth Evans, Andrew Shenton, Emma Woodward, Fiona Lalloo, Anthony Howell, Eamonn R Maher

**Affiliations:** 1 Academic Unit of Medical Genetics and Regional Genetics Service, St Mary's Hospital Manchester M13 0JH, UK; 2 Genesis Prevention Centre, Wythenshawe Hospital, Manchester M20, UK; 3 Section of Medical and Molecular Genetics, University of Birmingham School of Medicine, and West Midlands Regional Genetics Service, Birmingham, UK; 4 Department of Medical Oncology, Christie Hospital, Manchester M20 4BX, UK

## Abstract

**Background:**

The identification of a *BRCA1 *or *BRCA2 *mutation in familial breast cancer kindreds allows genetic testing of at risk relatives. However, considerable controversy exists regarding the cancer risks in women who test positive for the family mutation.

**Methods:**

We reviewed 385 unrelated families (223 with *BRCA1 *and 162 with *BRCA2 *mutations) ascertained through two regional cancer genetics services. We estimated the penetrance for both breast and ovarian cancer in female mutation carriers (904 proven mutation carriers – 1442 females in total assumed to carry the mutation) and also assessed the effect on penetrance of mutation position and birth cohort.

**Results:**

Breast cancer penetrance to 70 and to 80 years was 68% (95%CI 64.7–71.3%) and 79.5% (95%CI 75.5–83.5%) respectively for *BRCA1 *and 75% (95%CI 71.7–78.3%) and 88% (95%CI 85.3–91.7%) for *BRCA2*. Ovarian cancer risk to 70 and to 80 years was 60% (95%CI 65–71%) and 65% (95%CI 75–84%) for *BRCA1 *and 30% (95%CI 25.5–34.5%) and 37% (95%CI 31.5–42.5%) for *BRCA2*. These risks were borne out by a prospective study of cancer in the families and genetic testing of unaffected relatives. We also found evidence of a strong cohort effect with women born after 1940 having a cumulative risk of 22% for breast cancer by 40 years of age compared to 8% in women born before 1930 (p = 0.0005).

**Conclusion:**

In high-risk families, selected in a genetics service setting, women who test positive for the familial *BRCA1/BRCA2 *mutation are likely to have cumulative breast cancer risks in keeping with the estimates obtained originally from large families. This is particularly true for women born after 1940.

## Background

Since the identification of the *BRCA1 *[1] and *BRCA2 *[2] genes a great deal of debate has focussed on the issue of breast and ovarian cancer risk associated with mutations in these genes. It is clear that calculated cancer risks are dependent on the method of ascertainment of the families studied. Thus, breast cancer risks in large familial breast cancer kindreds with *BRCA1/BRCA2 *mutations are substantially higher than risks derived from population based studies [3,7,8]. In the high-risk families that recruited to the Breast Cancer Linkage Consortium (BCLC) cohort, *BRCA1 *and *BRCA2 *mutations were estimated to cause a cumulative lifetime risk of breast cancer at age 70 years of 85–87% and 77–84% respectively [3,7,8]. However, estimates of breast cancer risks to age 70 years of age derived from previous population based studies to date are much lower at 28–60% [4-6] for *BRCA1*, and lower still for *BRCA2*. It has been suggested that even these studies may overestimate the effect of the *BRCA1/2 *mutation alone [9]. Whilst there is some evidence of variation of cancer risk by position of mutation within each gene [10-12], more variation occurs between families with the same mutation. Therefore it is likely that a substantial proportion of the breast cancer risk in strong familial clusters with a *BRCA1/2 *mutation (the group of families that are usually seen by a Cancer Genetics Service), might be contributed to by modifier genes [13]. Optimum clinical practice requires, that the cancer risks provided to families undergoing genetic testing are appropriate to the setting in which the mutation was detected. To determine the most appropriate risks for women attending clinical cancer genetics services we determined the cumulative risks of breast and ovarian cancer for 385 families with pathogenic *BRCA1*/2 mutations identified in North West and Central England covering a population of 10 million.

## Methods

### Index cases and relatives

Breast and ovarian cancer families have been tested for *BRCA1/2 *mutations (using a whole gene analysis including a test for large deletions) since 1996 in the overlapping regions of Manchester and Birmingham in mid-north England. All genetic testing is undertaken with informed consent and consent is also taken to confirm cancer diagnosis. The study was carried out with Local Ethical committee approval. Women who attend the specialist genetic clinics in these regions with a family history of breast/ovarian cancer have a detailed family tree elicited with all first, second and if possible third degree relatives recorded. If a *BRCA1/2 *mutation is identified, further extensive attempts are made to ensure that all individuals at risk of inheriting the family mutation are represented on the pedigree. All cases of breast or abdominal cancers are confirmed by means of hospital/pathology records, from the Regional Cancer Registries (data available from 1960) or from death certification. Once a family specific pathogenic *BRCA1/2 *mutation is identified predictive testing is offered to all blood relatives. Where possible all affected women with breast/ovarian cancer are tested to establish the true extent of *BRCA1/2 *involvement in the family. In many large families it is possible to establish "obligate" gene carriers by testing for the same mutation in different branches of the family, thereby establishing that intervening relatives carry the same mutation.

All female *BRCA1/2 *mutation carriers identified were included in this study, and their details, those of all tested relatives and first-degree untested female relatives were entered onto a Filemaker Pro 5 database. The initial individual in which a mutation was identified was designated the "index" case, with all other individuals being classified as to their position in the pedigree compared to a proven mutation carrier. All women reaching 20 years were entered if untested for a mutation. The exception was mothers of a mutation carrier when it was clear that the mutation was paternally inherited. 385 index cases were studied and from these extended pedigrees information on a total of 2466 females was collected. Information was entered on date of birth, date of last follow up, breast cancer status, ovarian cancer status, dates of diagnoses and date of death (if applicable), gene mutation carried in the family, their relationship to a known mutation carrier and their mutation status and date at which testing took place.

The proportions of unaffected first-degree relatives (FDRs) testing positive or negative was derived for each age cohort. Figures from this were used to estimate the proportion of untested relatives that were likely to test positive in each age group. The proportion of untested FDRs with breast or ovarian cancer that were likely to test positive was similarly estimated from testing that had taken place in each family. Penetrance analysis was performed by including all mutation positive individuals and appropriate numbers of untested FDRs on a proportional basis. Kaplan Meier curves were derived for breast and ovarian cancer incidence for each gene and by dividing each gene into the previously identified ovarian cancer cluster region (OCCR): exon 11 (nucleotides 2401–4190) for *BRCA1 *and exon 11 (nucleotides 3035–6629) for *BRCA2*. For *BRCA1 *we used the nucleotide range identified by the BCLC [11], although this is not traditionally called an OCCR it is the region published as having the greatest proportional risk of ovarian cancer. Individuals were censored at age of death, age of last follow up, age at appropriate cancer or age at appropriate risk reducing surgery (oophorectomy for ovarian cancer, mastectomy and oophorectomy for breast cancer). The Manchester scoring system was used to assess the strength of the breast/ovarian cancer history [14]. This system was devised to assess the likelihood of a BRCA1/2 mutation and scores breast and ovarian cancers individually in the family, giving a higher score the younger the age at diagnosis [14]. A combined score of 20 reflects a 20% likelihood of identifying a BRCA1/2 mutation.

## Results

The 385 families consisted of 223 apparently unrelated *BRCA1 *and 162 *BRCA2 *families. Mutations were spread throughout the *BRCA1 *and *BRCA2 *genes with the commonest mutation being the Jewish exon 2 185 DelAG (31 families). There were also 20 families with single or multiple exon deletions or duplications in *BRCA1 *and 6 in *BRCA2*. These families contained 904 proven female mutation carriers (526 in *BRCA1*; 378 in *BRCA2*). There were 992 female FDRs of unknown mutation status: 554 in *BRCA1*; 438 in *BRCA2 *kindreds. Of these 244 had been diagnosed with breast cancer, 88 with ovarian cancer and 14 with both. 21/206 (10%) FDRs with breast cancer tested negative for the family mutation, but only 1/101 FDRs with ovarian cancer. The age distribution of the breast cancer cases testing negative for the mutation was identical to those testing positive. We therefore assumed that every tenth untested FDR with breast cancer (only) was negative for the mutation in each gene. All 21 individuals testing negative for the family mutation were also negative for the 1100delC mutation in CHEK2. As 99% of the ovarian cancers tested were positive we assumed that all FDRs with ovarian cancer were positive. The results for predictive testing of unaffected females for each gene are shown in Table 1. We assumed that similar proportions of untested unaffected female relatives would test positive for each gene. We therefore stratified these relatives by age and excluded an increasing proportion of the relatives as indicated for each age group. For the age group of 60 years and over we assumed that 10% would be positive for *BRCA1 *and 20% for *BRCA2*.

**Table 1:** Proportion of living unaffected FDR females undertaking presymptomatic predictive genetic testing by gene and age cohort.

Predictive test result By age	BRCA1 +ve	BRCA1-ve	Number positive BRCA1	BRCA1 untested	BRCA2 +ve	BRCA2-ve	Number positive BRCA2	BRCA2 untested	Proportion positive BRCA1 assumed	Proportion positive BRCA2 assumed
18–30 yrs	28	34	28/62 (45%)	41	18	19	18/37 (48%)	34	50%	50%
30–39 yrs	51	42	51/93 (55%)	78	50	41	50/91 (55%)	64	50%	50%
40–49	22	44	22/66 (33%)	57	34	27	34/61 (56%)	49	33%	50%
50–59	10	19	10/29 (34%)	43	14	21	14/35 (40%)	59	33%	40%
60+	2	24	2/26 (8%)	60	4	19	4/23 (17%)	69	10%	20%
Total	113	163	103/266	279	110	127	110/240	247		

The proportion testing positive for each gene with Manchester scores [14] above and below 20 and 23 are presented in Table 2. This shows a substantial effect of cancer burden for *BRCA2 *with high-risk families (scores above 20 points) having a much lower proportion of positive predictive tests after 50 years.

**Table 2:** Proportion of predictive tests positive in unaffected FDR women >50 years of age by family Manchester score for each gene

Manch score predictives	Above or =	Below
(score)	% positive	% positive
BRCA2 >50 (23)	9/37 (24%)	9/24 (37.5%)
BRCA2 >50 (20)	10/44 (23%)*	8/17 (47%)*
BRCA1 >50 (23)	7/33 (21%)	5/20 (20%)
BRCA1 >50 (20)	8/39 (20.5%)	4/14 (29%)

*chi square for difference 3.48; p = 0.07

**Figure 1:**
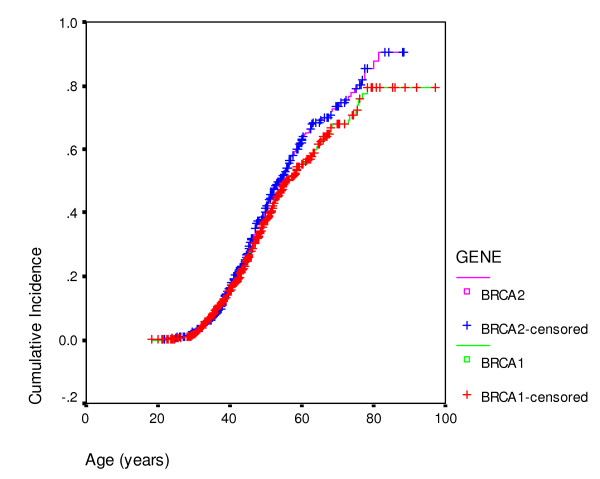
Breast cancer cumulative incidence by gene (*BRCA1 or BRCA2*).

**Figure 2:**
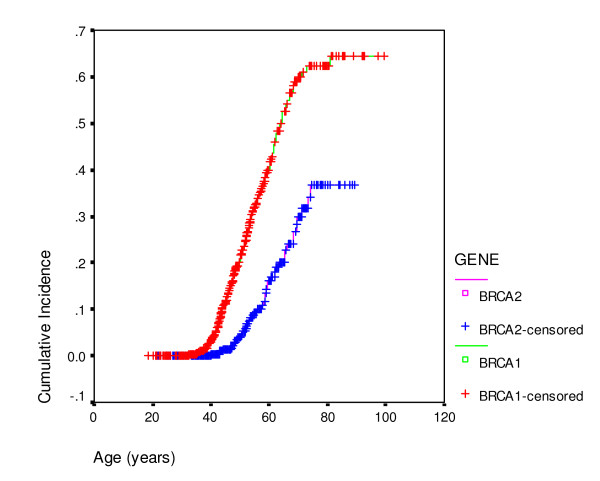
Ovarian cancer cumulative incidence by gene (*BRCA1 or BRCA2*).

Overall, of the FDRs of unknown mutation status, 92/92 with ovarian cancer, 220/244 with breast cancer and 234/648 unaffected FDRs were included in the analysis. In total this amounted to 839 actual and presumed carriers for *BRCA1 *and 603 actual and presumed carriers for *BRCA2*. There were 243/839 (29%) *BRCA1 *individuals with ovarian cancer compared to 64/603 (11%) female *BRCA2 *carriers. 411/839 (49%) *BRCA1 *carriers and 355/603 (59%) *BRCA2 *carriers had developed breast cancer. Penetrance estimates for each gene are shown (Table 3; Figures 1, 2) for breast and ovarian cancer. The curves were remarkably similar for each gene, with breast cancer penetrance to 70 and 80 years of 68% (95%CI 65–71%) and 79.5% (95%CI 75–84%) for *BRCA1 *and 74% (95%CI 71–77%) and 88% (95%CI 85–91%) for *BRCA2*. Ovarian cancer risk to 70 and 80 years was 60% (95%CI 65–71%) and 65% (95%CI 75–84%) for *BRCA1 *and 30% (95%CI 25.5–34.5%) and 37% (95%CI 31.5–42.5%) for *BRCA2*. The penetrance for ovarian cancer was significantly higher for *BRCA1 *(p < 0.0001), but breast cancer incidence for *BRCA2 *was borderline significantly higher than for *BRCA1 *(p = 0.09). Indeed breast cancer penetrance estimates for *BRCA2 *after 60 years were significantly higher as was overall penetrance including the index case (p = 0.02). There was no significant effect of ovarian cancer cluster regions (OCCR, nucleotides 2401–4190 *BRCA1 *and nucleotides 3035–6629 in *BRCA2*) for either gene with lifetime ovarian cancer risks (to 80 years) of 65% for 573 *BRCA1 *carriers outside the OCCR and 70% for 266 women with mutations within the OCCR (p = 0.18). Similarly there was no effect of position for *BRCA2 *with lifetime risks of 37% for 373 *BRCA2 *carriers outside the OCCR and 41% for 230 *BRCA2 *women with OCCR mutations (p = 0.17). There was a 10% higher cumulative incidence at most ages for breast cancer in those outside the *BRCA2 *OCCR, although lifetime risk was little different at 90% and statistical significance was not reached (p = 0.07). No such difference was seen for *BRCA1 *with virtually identical incidence curves (p = 0.25). DCIS was included as breast cancer. However, this only amounts to 1% of *BRCA1 *breast cancers and 2% for *BRCA2*. It is likely that nearly all of these would have become invasive as only 1/16 occurred after 60 years of age. Tamoxifen is not licensed in the UK for prevention. Only 23 mutation carriers took tamoxifen as part of the IBIS1 prevention trial and this is unlikely to have materially changed the penetrance estimates.

**Table 3:** Penetrance for breast and ovarian cancer by age for BRCA1 and BRCA2.

Cancer risk to age	BRCA1 Breast (se)	BRCA2 Breast (se)	BRCA1 Ovary (se)	BRCA2 Ovary (se)
30	2%	2.5%	0	0
40	16.5%(0.015)	17%(0.019)	3% (0.007)	0
50	48%(0.023)	42%(0.027)	21%(0.02)	4% (0.012)
60	55%(0.027)	63%(0.031)	40%(0.024)	16% (0.03)
70	68% (0.033)	75%(0.033)	60% (0.037)	30% (0.046)
80	79.5% (0.04)	88%(0.037)	65% (0.042)	37% (0.056)

se-standard error

**Figure 3:**
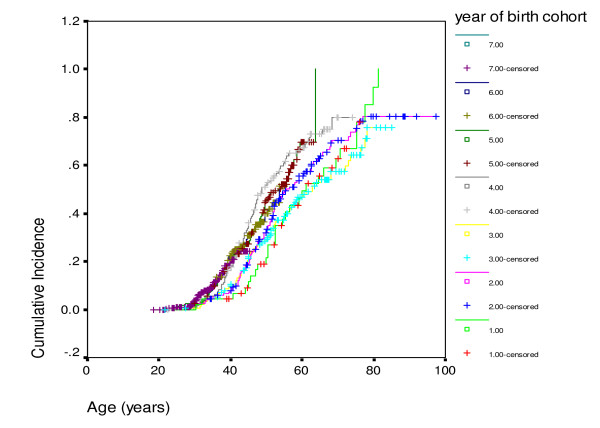
**Cumulative risk of breast cancer by age cohort for *BRCA1 *and *BRCA2 *combined after exclusion of the index case.** Risk to 40 years: Group 1 (birth year <1900; n = 45) 4%; Group 2 (1900–1920; n = 154) 8%; Group 3 (1920–1930; n = 154) 10%; Group 4 (1930–1940; n = 124) 17%; Group 5 (1940–1950; n = 162) 21%; Group 6 (1950–1960; n = 265) 23%; Group 7 (1960+; n = 276) 22%. Log rank (df 6) 153; p = 0.0005.

An estimate for breast cancer penetrance was also made for each 10–20 year birth cohort. A highly significant difference was identified with those born after 1960 having a breast cancer risk to 40 years of age of 40% compared to only 7.5% for those with a year of birth between 1900 and 1920 (Figure 3: p < 0.00001). However, after exclusion of the index case the cumulative risk to 40 years dropped to between 21–23% for the birth cohorts after 1940. This was, nonetheless still a highly significant trend (p = 0.0005). After exclusion of the index case there was no significant birth cohort effect observed for ovarian cancer (p = 0.086). To assess the earlier age at breast cancer diagnosis on life expectancy we carried out a Kaplan-Meier survival analysis on the birth cohorts, again excluding the index case. There was no significant difference in survival from birth (Table 4; log rank df 6, p = 0.07), although there was a trend to better survival in the earlier birth cohorts. Indeed if the index case was included 21/83 (25%) index cases post birth year 1960 had died by 45 years of age, equivalent to a cumulative mortality of 35% to that age.

**Table 4:** Survival analysis from birth, *BRCA1 *and *BRCA2 *combined for each birth cohort, index case excluded

% died from birth to age	<1900 (n = 45)	1900–1919 (n = 154)	1920–1929 (n = 154)	1930–1939 (n = 124)	1940–1949 (n = 158)	1950–1959 (n = 162)	1960+ (n = 276)
45 years	15%	14%	17%	17%	21%	15%	17%
60 years	42%	56%	57%	62%	56%		
70 years	73%	74%	77%	85%			

Log Rank 11.53 df6 p = 0.07

Breast cancer incidence was also assessed after family ascertainment. Incidence figures for breast/ovarian cancer are shown in Table 5. These reflect the incidence in unaffected women at the time of family ascertainment and follow up was censored at the time of risk reducing surgery (oophorectomy/mastectomy). As the index case was used to identify the mutation usually on surveillance the incidence rates for these cases are artificially high. Excluding the index cases there was an incidence of 2.5–2.7 per thousand for breast cancer in proven carriers. Even including 40% of the follow up time and 80% of the breast cancers from the FDR unknown category (Tables 1 and 2), this still gave an annual incidence of breast cancer of 1.98% for both *BRCA1/2 *mutation carriers (38/1917; 35/1763.6). An annual rate of 2% averaged over the risk period of 30–79 years would if anything indicate a higher risk than those indicated by the Kaplan-Meier analysis.

**Table 5:** Breast and ovarian cancers occurring after the family was referred to the genetics centre.

	Number of women	Years f/u (Breast ca)	Cancers	Rate (%/Year)	Number of women	Years f/u (ovarian ca)	Cancers	Rate
*BRCA1 *Carriers	256	1522.33	65	4.27	403	2256.13	37	1.64
*BRCA1 *FDR unknown	341	1988.07	13	0.65	355	2049.42	6	0.29
*BRCA1 *Index	87	399.49	37	9.3	194	977.35	24	2.5
*BRCA1 *Carriers less index	159	1122.33	28	2.5	209	1278.78	13	1.01
*BRCA2 *Carriers	210	1210.41	61	5.0	363	2235.14	12	0.54
*BRCA2 *FDR unknown	324	2096.93	12	0.57	345	2187.04	4	0.19
*BRCA2 *Index	66	285.59	36	12.6	175	966.69	9	0.93
*BRCA2 *Carriers less index	144	924.82	25	2.7	363	1268.45	3	0.24

Censored if affected with relevant cancer prior to date of referral and at RRS, relevant cancer diagnosis, or date of last follow up.

The index case was used to identify the mutation. Women were censored at time of risk reducing surgery.

## Discussion

We present data on a large cohort of women identified as carriers or presumed carriers of *BRCA1 *and *BRCA2 *mutations in a large proportion of the UK population. The penetrance estimates derived from these women are very similar to those derived from the BCLC cohort of high-risk families with lifetime risks of breast cancer of close to 85% for both genes [3,7,8]. The estimate of ovarian cancer was also very similar with risks to 70 years of 60% for *BRCA1 *carriers and 33% as opposed to 27% [3] for *BRCA2 *carriers. It is possible that the higher overall breast cancer estimates for *BRCA2 *were related to competing mortality from ovarian cancer. Many risk factors for breast and ovarian cancer are similar (early menarche, late menopause, nulliparity) and women with these may have died from ovarian cancer before they developed breast cancer. This effect would be more prominent for *BRCA1 *and would potentially explain the higher breast cancer penetrance for *BRCA2*. The ratio of those testing positive:negative for the BRCA mutation whilst still unaffected also gives support to high penetrance. Of those women without an affected daughter, <10% of those aged over 60 years, tested positive for *BRCA1 *and <20% for *BRCA2*. The figures over 60 years are, nonetheless based on small numbers. The earlier drop in positive:negative ratio for *BRCA1 *almost certainly represents a higher combined risk of both breast and ovarian cancer to 50 and 60 years. Another supportive feature is shown in Table 2. The typical families tested in our centre have a Manchester score of 20+ reflecting multiple early onset breast and/or ovarian cancer in the family. The less "high" risk clusters as evidenced by lower Manchester scores had a higher proportion testing positive >50 years. This suggests that Manchester score could be used as a bench-mark to predict penetrance particularly in *BRCA2 *families. Whilst all attempts to assess penetrance have their inherent biases and assumptions this cannot be said of the results of presymptomatic testing. The only potential bias would be if women had an inkling that they would test positive or negative prior to coming forward. This is not borne out by our results particularly accounting for Manchester score.

The previously reported positional effect of mutations for both *BRCA1 *and *BRCA2 *is not borne out by our analysis. No substantial effect of increased risk of ovarian cancer was seen in the respective ovarian cluster regions of each gene and only a borderline significant reduction of breast cancer risk was seen for *BRCA2*. Much of the OCCR association has been based on ratios of breast to ovarian cancer [10] or on the presence or not of ovarian cancer in the family [11]. Even this reliance on the presence of ovarian cancer for *BRCA2 *has been questioned by the report of 58% of *BRCA2 *related ovarian cancer families having mutations outside the OCCR [12]. Although the BCLC study on *BRCA1 *positional effect [10] included 356 families compared to our 223 families no absolute estimate of penetrance was made. Whilst the breast cancer incidence was lower in the central portion of the gene (nucleotides 2401–4190) (RR 0.71) in their analysis it was not possible to derive absolute risk figures for each portion of the gene. Additionally it is likely that our more extensive testing of unaffected relatives may provide a more accurate overall picture as reported here. Accurate estimates of cancer risk are essential for families and individuals undertaking genetic testing. Based on our analysis, it is questionable whether any account should be taken of the OCCR in each gene or indeed any substantial positional effect in genetic counselling.

It is also clear that for individuals undertaking predictive genetic testing in the context of families ascertained from cancer genetic clinics as opposed to population testing that risk figures similar to those derived in our study or the BCLC is quoted in our own clinics and we recommend that penetrance estimates are derived for the population being counselled. Our data are nonetheless at variance to a similar analysis carried out in North America [15]. A series of 1948 families were tested for mutations in *BRCA1*/2 in eight centres. 283 families with *BRCA1 *mutations were identified and 143 in *BRCA2*. The authors used statistical modelling to arrive at penetrance figures by 70 years of 46% (95%CI 39–54%) for *BRCA1 *and 43% (95%CI 36–51%) for *BRCA2*. The authors did not appear to take advantage of any further testing of relatives in the family. Whilst they corrected for potential ascertainment bias, they did not allow for the effects of modifier genes in these families and purely looked at attributable risk from *BRCA1 *and *BRCA2 *mutations alone. This was based on the apparent lack of heterogeneity in another study of Jewish families from North America [16]. What is particularly concerning is the risk attributed to "non mutation carriers" to 70 years. A figure of 5% as a general population risk for breast cancer may have been correct 20–30 years ago, but is certainly not the risk faced by women in the US or the UK today. Breast cancer risk to age 70 is 7.6% in the UK [17] and nearer 8% in the US. A correction for this difference might give penetrance figures of nearer 74% for *BRCA1 *and 69% for *BRCA2*. The decision not to include any adjustment in these families for the effects of modifier genes is questionable. The difference in penetrance obtained from the BCLC and from population studies strongly suggests the presence of additional genetic factors in high-risk families. We have recently reported that those testing negative for a family BRCA mutation are still at 3-fold relative risk of breast cancer [13]. This phenocopy effect was also seen in the Iceland data for their founder *BRCA2 *mutation, although to a lesser extent given the strong population based element of their analysis [18]. However, it is possible that modifier genes are more prevalent in some populations and that penetrance in North America is less affected by modifier genes than in the UK. The presence of these modifier alleles is now indisputable from recent genome wide association studies [19-21].

A potential criticism of our study is that we have not taken enough account of ascertainment bias and that additional adjustment maybe necessary beyond excluding the index case. An analysis using these adjustments was carried out in the North American study [15] and recent reports from the Cambridge group [22]. These studies did not take into account the widespread testing of relatives and as explained above the American study deliberately excluded any effect other than of the *BRCA1/2 *mutation. Whilst it is clearly interesting to know the effect of *BRCA1/2 *alone, women undergoing testing will want to know what their own specific risk of breast and ovarian cancer are, including that contributed by other potential "modifier" genes in their family. We must also acknowledge that confidence intervals in table 3 should also be wider due to forcing the data on unknown FDRs into a known category.

The high-risk women testing positive is also supported by the prospective part of our study. The 2–2.7% annual risk demonstrated is equivalent to the highest risk in a 10-year period (23% *BRCA1*; 30% *BRCA2*-Table 3). Although most of the breast cancers were detected by screening, only one was detected at a prevalence mammogram. These follow up risks are also supported by a similar follow up study in the Netherlands where 8 breast cancers occurred in 63 mutation carriers with a calculated annual risk of 2.5% [23].

Our own study and recent analyses from North America and Iceland demonstrate that women in the most recent birth cohort have a substantially higher risk of developing breast cancer than past cohorts [16,18]. The incidence of breast cancer in *BRCA2 *carriers has risen 4 fold in 80 years in Iceland (as has breast cancer in the general population) and we have observed a similar increase from <10% risk by 40 years in those born before 1930 to a 40% risk on those born after 1960, although this was less significant after allowing for ascertainment bias. It is, therefore, inappropriate to quote risks as low as 43–46% (based on population studies) for lifetime breast cancer risk to women in their twenties or early thirties if they test positive for a mutation in a high-risk family. Another potential effect of earlier breast cancer might be a reduction in life expectancy. With increasing survival from birth in the general population and improved survival from diagnosis of breast cancer we might have expected to see improved life expectancy. However, it would appear that these elements almost completely cancel each other out and there is no evidence for improved survival from birth in modern BRCA birth cohorts.

When discussing the higher risks of breast cancer in recent generations, it is nonetheless important to couch any discussion on risk in terms of future prospects for risk reduction by preventive measures. Increasing numbers of women are opting for risk reducing surgery particularly early RRO, which will substantially reduce the risk of both breast and ovarian cancer [24]. It is also likely that new treatments or substantial changes from the Western lifestyle may have a sufficient effect to help in risk management in the future.

## Conclusion

We believe our results show that when counselling women on their risks of breast and ovarian cancer if they carry a family *BRCA1/2 *mutation the risks should reflect the context of cancer in their family and not just an average risk from possibly over-corrected penetrance estimates from population studies. Indeed a recent review in a prestige journal quoted "headline" risks for *BRCA2 *of only 40% and 8% for breast and ovarian cancer to 80 years [25]. Understandably many clinicians and counsellors may quote these risks. The use of family cancer burden in adjusting risks to carriers is already used in the BOADICEA programme [26] and the Manchester score could also be used as a bench mark of where in the range of 40–90% breast cancer risk a women should be steered, especially for *BRCA2*.

## Competing interests

The authors declare that they have no competing interests.

## Authors' contributions

DGE: Conception. DGE, AS, EW, FL: Data collection. DGE and AS: Data analysis. DGE, AS, FL, AH, ERM: Manuscript writing. All authors read and approved the final version of the manuscript.

## Pre-publication history

The pre-publication history for this paper can be accessed here:


